# *Cosmos Caudatus* as a Potential Source of Polyphenolic Compounds: Optimisation of Oven Drying Conditions and Characterisation of Its Functional Properties 

**DOI:** 10.3390/molecules180910452

**Published:** 2013-08-29

**Authors:** Ahmed Mediani, Faridah Abas, Alfi Khatib, Chin Ping Tan

**Affiliations:** 1 Department of Food Science, Faculty of Food Science and Technology, Universiti Putra Malaysia, 43400 Serdang, Selangor, Malaysia; E-Mails: medianiahmed47@gmail.com (A.M.); faridah@food.upm.edu.my (F.A.); 2 Laboratory of Natural Products, Institute of Bioscience, Universiti Putra Malaysia, 43400 Serdang, Selangor, Malaysia; 3 Faculty of Pharmacy, International Islamic University Malaysia, 25200 Kuantan, Pahang, Malaysia; E-Mail: alfikhatib1971@gmail.com; 4 Department of Food Technology, Faculty of Food Science and Technology, Universiti Putra Malaysia, 43400 Serdang Selangor, Malaysia

**Keywords:** *Cosmos caudatus*, antioxidant capacity, RSM, optimization, polyphenolic compounds, oven drying

## Abstract

The aim of the study was to analyze the influence of oven thermal processing of *Cosmos caudatus* on the total polyphenolic content (TPC) and antioxidant capacity (DPPH) of two different solvent extracts (80% methanol, and 80% ethanol). Sonication was used to extract bioactive compounds from this herb. The results showed that the optimised conditions for the oven drying method for 80% methanol and 80% ethanol were 44.5 °C for 4 h with an IC_50_ of 0.045 mg/mL and 43.12 °C for 4.05 h with an IC_50_ of 0.055 mg/mL, respectively. The predicted values for TPC under the optimised conditions for 80% methanol and 80% ethanol were 16.5 and15.8 mg GAE/100 g DW, respectively. The results obtained from this study demonstrate that *Cosmos caudatus* can be used as a potential source of antioxidants for food and medicinal applications.

## 1. Introduction

Almost all herbs are fragile when they are in their fresh state and can easily deteriorate in a short time after harvest. For this reason, drying is the best way to preserve the efficiency of their metabolites for a long period of time [1,2]. Herbs have been gaining attention in many countries because of the medicinal and nutritional benefits provided by their potential natural antioxidant properties. According to the World Health Organization (WHO), more than three of four inhabitants of Asian and African countries utilise natural herbs as replacements for synthetic medicines [3]. Hence, phytomedicines or herbal medicine products constitute beneficial business throughout the World [3]. The use of herbs as antioxidants in food products has also been given much emphasis and attention in replacing artificial antioxidants in human diets [4,5,6,7]. The success of Malaysian herb therapies have emphasised the great potential for Malaysian herbs to find widespread use in the treatment of numerous diseases. For instance, ‘*kesum*’ (*Persicaria hydropiper*), ‘*selom*’ (*Oenanthe javanica*), ‘*pegaga*’ (*Centella asiatica*), ‘curry leaf’ (*Murraya koenigii*) and *Cosmos caudatus* are used to prevent cancer. 

*Cosmos caudatus*, which is locally known as *Ulam raja*, is a herb of the family Compositae and is a common and popular traditional vegetable that is often consumed raw as a salad. Use of this herb, which originated from tropical Central America, is widespread in all regions of Malaysia [8]. It is eaten raw due to its unique, appealing smell and aroma, which add diversity and taste to food [8,9]. It is also used as food flavouring and in traditional medicine. In addition, some nutritional and medicinal studies have demonstrated that *Ulam raja* is a rich source of bioactive compounds including phenolics, flavonoids, carbohydrates, proteins, minerals and vitamins, increasing its nutritional value [9]. In the last decade, interest in the medicinal and nutritional applications of this herb has increased dramatically. Due to its functional properties, it has also been demonstrated to support the bone formation and aid blood circulation [9]. However, knowledge of its true benefits to confirm the vast traditional utilisation is lacking; people believe in the efficiency of its metabolites, creating a need for supplementary research. Few phytochemical compounds have been reported for this herb, and little research has been conducted on its antioxidative efficacy and total phenolic content (TPC). Therefore, this herb was selected for this study. 

Phenolic compounds are the most widely active groups of antioxidant agents and sources of natural antioxidants. Phenolic compounds are important constituents of fruits, vegetable and herbs and are better antioxidant than vitamins C and E and β-carotene. They can play a key role in scavenging free radicals that cause oxidative stress because some phenolic components have substantial antioxidant capacity against peroxyl radicals. In addition, phenolic compounds have been shown to possess potential antioxidant ability, which helps them to scavenge electrophiles and active oxygen species, slow down nitrosation and chelate metal ions to limit auto-oxidation and increase the ability to adjust some enzyme actions [2,10]. Several studies have reported a decline in the TPC and antioxidant capacity of herbs after thermal processing methods [11,12,13,14,15]. Phytochemical compounds are sensitive to thermal processing methods, and thus the processing conditions should be optimised for preserving their benefits in the final food product [16]. These conditions, including drying time and temperature, tend to have considerable influences on the decline of the bioactive compound efficiency of the final products [16]. 

Drying can cause substantial changes in the physical and organoleptic properties of dried foods [1,2]. Several changes to food occur when it is subjected to this processing method. There is increasing concern that some thermal methods negatively affect the bioactive components of herbs. Oven drying is considered one of the easiest and most rapid thermal processing methods that can preserve phytochemical content. Numerous studies have been performed to study the influence of oven drying on phytochemical compounds of herbs [17]. For economic reasons, processing time and the ability to preserve the sample during processing have gained substantial attention from many researchers. However, only limited knowledge is available for the optimisation of drying conditions (time and temperature), which might provide a key solution for preserving herbs in a short period of time with low cost or, at a minimum reducing the thermal drying effects on herbs. The presence of natural antioxidants in some herbs that are traditionally believed to have antioxidant capacity compels researchers to attempt to identify appropriate methods for protecting herb phytochemicals. Therefore, this paper aimed to optimise oven drying conditions (time and temperature) to preserve the constituents or at least minimise the effect of this processing method on antioxidant capacity and total phenol content of *C. caudatus* extracted with two different solvents (80%, methanol and 80% ethanol) by using response surface methodology (RSM).

## 2. Results and Discussion

### 2.1. Radical Scavenging Activity (DPPH)

As shown in Table 1, the air-dried *C. caudatus* control sample that was extracted with 80% methanol or 80% ethanol possessed high free radical scavenging activity, with IC_50_ values of 29 and 32 µg/mL, respectively. The IC_50_ for the oven-dried samples ranged between 47–142 µg/mL and 54–145 µg/mL for extraction with 80% methanol or 80% ethanol, respectively. The control result was in close agreement with that reported by Mustapha *et al*. [18], who reported that the IC_50_ value of *C. caudatus* was 21.31 µg/mL. Moreover, oven drying resulted in a significant reduction in DPPH radical scavenging activity compared to air drying. This decrease in scavenging ability could be due to the effect of thermal heating upon the drying processing period. In addition, drying temperature might cause the degradation of the metabolites responsible for the antioxidant capacity of the herbs, such as vitamins C and A, phenolic compounds and carotenoids, via chemical reactions such as the formation of polymerization products or their rearrangements. The statistical analysis results from Table 2 and Table 3 yield the following predicted equations for the IC_50_ for the samples extracted with 80% methanol and 80% ethanol, respectively:

Y_1_ = 0.084885 + 0.020503X_1_ + 0.028880X_2_ + 0.005077X_2_^2^ + 0.007000X_1_X_2_ (1)

Y_3_ = 0.093286 + 0.017098X_1_ + 0.027931X_2_ + 0.011750X_1_X_2_ (2)

### 2.2. Total Phenolic Content

As shown in Table 1, the TPC values of the control air-dried *C. caudatus* sample extracted with 80% methanol and 80% ethanol were higher than those of the oven-dried samples: 22.3 and 19.3 g GAE/100 g DW, respectively. However, the findings of the current study did not support those of a previous study [18] that reported a value of 704.21 mg GAE/g for this sample. This difference may be due variations in the methods used, the sample conditions and harvesting time. The TPC value decreased significantly (*p* < 0.05) in the oven-dried samples compared with the control, with values of 5.84–16.25 for 80% methanol and 5.34–14.94 for 80% ethanol. This reduction is likely due to the effect of temperature on the stability of TPC. Thermal processing might cause stress to the herb, destroying the cell wall and resulting in the degradation of TPC compounds, while also increasing the sensitivity of these components to oxidative stress. The statistical analysis results from Table 2 and Table 3 yielded the following predicted equations for the TPC for the 80% methanol and 80% sample ethanol extracts, respectively:

Y_2_ = 11.3036 – 1.3372X_1_ – 3.3328 X_2_ – 0.8600X_1_X_2_ (3)

Y_4_ = 10.3533 – 1.3952X_1_ – 2.8987X_2_ +0.9821X_1_^2^ – 0.7754X_2_^2^ (4)

**Table 1:** RSM CCD cubic design and response variables (IC_50_ and TPC) for 80% methanol and 80% ethanol.

Run Order	Point Type	Blocks	Drying Time	Oven Temperature	80% Methanol		80% Ethanol	
					IC_50_ (mg/mL) Y_1_	TPC (g GAE/100g DW) Y_2_	IC_50_ (mg/mL) Y_3_	TPC (g GAE/100g DW) Y_4_
control					0.029 ± 0.01	22.3 ± 0.96	0.032 ± 0.02	19.3 ± 0.80
1	1	1	9.00	50.00	0.074 ± 0.01	13.82 ± 0.13	0.072 ± 0.02	12.65 ± 0.40
2(c)	0	1	6.50	70.00	0.088 ± 0.03	11.70 ± 0.12	0.084 ± 0.06	10.75 ± 0.30
3(c)	0	1	6.50	70.00	0.082 ± 0.02	11.87 ± 0.25	0.087 ± 0.03	10.10 ± 0.40
4	1	1	4.00	90.00	0.087 ± 0.02	10.13 ± 0.34	0.092 ± 0.05	8.75 ± 0.50
5	1	1	9.00	90.00	0.142 ± 0.01	5.84 ± 0.32	0.145 ± 0.07	5.48 ± 0.40
6(c)	0	1	6.50	70.00	0.078 ± 0.02	11.32 ± 0.11	0.095 ± 0.04	10.43 ± 0.30
7	1	1	4.00	50.00	0.047 ± 0.04	14.67 ± 0.15	0.066 ± 0.03	14.05 ± 0.40
8	−1	2	10.04	70.00	0.116 ± 0.02	10.32 ± 0.10	0.122 ± 0.01	10.35 ± 0.20
9(c)	0	2	6.50	70.00	0.088 ± 0.03	10.82 ± 0.30	0.092 ± 0.06	9.85 ± 0.20
10	−1	2	2.96	70.00	0.058 ± 0.03	14.25 ± 0.17	0.067 ± 0.01	14.94 ± 0.40
11	−1	2	6.50	41.76	0.054 ± 0.04	16.25 ± 0.13	0.054 ± 0.01	12.92 ± 0.20
12	−1	2	6.50	98.28	0.141 ± 0.05	6.25 ± 0.10	0.142 ± 0.01	5.34 ± 0.30
13(c)	0	2	6.50	70.00	0.091 ± 0.01	10.43 ± 0.14	0.095 ± 0.02	10.43 ± 0.40
14(c)	0	2	6.50	70.00	0.083 ± 0.02	10.58 ± 0.10	0.093 ± 0.05	10.56 ± 0.30

Note: (c) center point. Represented values are the mean and standard deviation of triplicate.

### 2.3. Effect of the Solvent on IC_50_ and TPC

The results showed that the IC_50_ and TPC values of the same conditions with both solvents varied relative to the result in Table 1. These variations are apparent in the runs as well as in the control samples. Furthermore, the 80% methanol extract exhibited superior values for both responses. Although the two sample t-test indicated that these results were not statistically significant for TPC, there was a significant effect on the TPC and IC_50_ values of the air-dried samples. These differences can be explained in part by the differences in the polarity of the two solvents and the compounds that contribute to the antioxidant capacity of *C. caudatus*.

**Table 2:** Regression coefficient parameters of samples extracted with 80% methanol.

Regression Coefficient	Symbol	Full Model				Reduce Model			
		Parameter Estimate		*p*- value		Parameter Estimate		*p*- value	
		IC_50_(Y_1_)	TPC(Y_2_)	IC_50_(Y_1_)	TPC(Y_2_)	IC_50_(Y_1_)	TPC(Y_2_)	IC_50_(Y_1_)	TPC(Y_2_)
Linear	X_1_	0.020503	−1.3372	0.000	0.001	0.020503	−1.3372	0.000	0.000
	X_2_	0.028880	−3.3328	0.000	0.000	0.028880	−3.3328	0.000	0.000
Quadratic	X^2^_1_	−0.000187	0.4194	0.902	0.124	-	-	-	-
	X^2^_2_	0.005062	−0.0981	0.011	0.695	0.005077	-	0.006	-
Interaction	X_1_X_2_	0.007000	−0.8600	0.010	0.034	0.007000	−0.8600	0.006	0.036
*R^2^*		98.9%	97.3%			98.9%	96.0%		
*R^2^* adjusted		98.0%	95.0%			98.3%	94.2%		

Note: X_1_: drying time; X_2_: oven temperature

**Table 3:** Regression coefficient parameters of samples extracted with 80% ethanol.

Regression Coefficient	Symbol	Full Model				Reduce Model			
		Parameter Estimate		*p*-value		Parameter Estimate		*p*-value	
		IC_50_ (Y_3_)	TPC (Y_4_)	IC_50_(Y_3_)	TPC(Y_4_)	IC_50_(Y_3_)	TPC(Y_4_)	IC_50_(Y_3_)	TPC(Y_4_)
Linear	X_1_	0.017098	−1.3952	0.000	0.000	0.017098	−1.3952	0.000	0.000
	X_2_	0.027931	−2.8987	0.000	0.000	0.027931	−2.8987	0.000	0.000
Quadratic	X^2^_1_	0.001125	0.9821	0.584	0.001	-	0.9821	-	0.002
	X^2^_2_	0.002875	−0.7754	0.186	0.005	-	−0.7754	-	0.007
Interaction	X_1_X_2_	0.011750	−0.4675	0.003	0.111	0.011750	-	0.002	-
*R^2^*		97.9	98.1			97.2	97.2		
*R^2^* adjusted		96.1	96.5			95.9	95.5		

X_1_: drying time; X_2_: oven temperature.

### 2.4. Effect of Independent Variables

**Figure 1:**
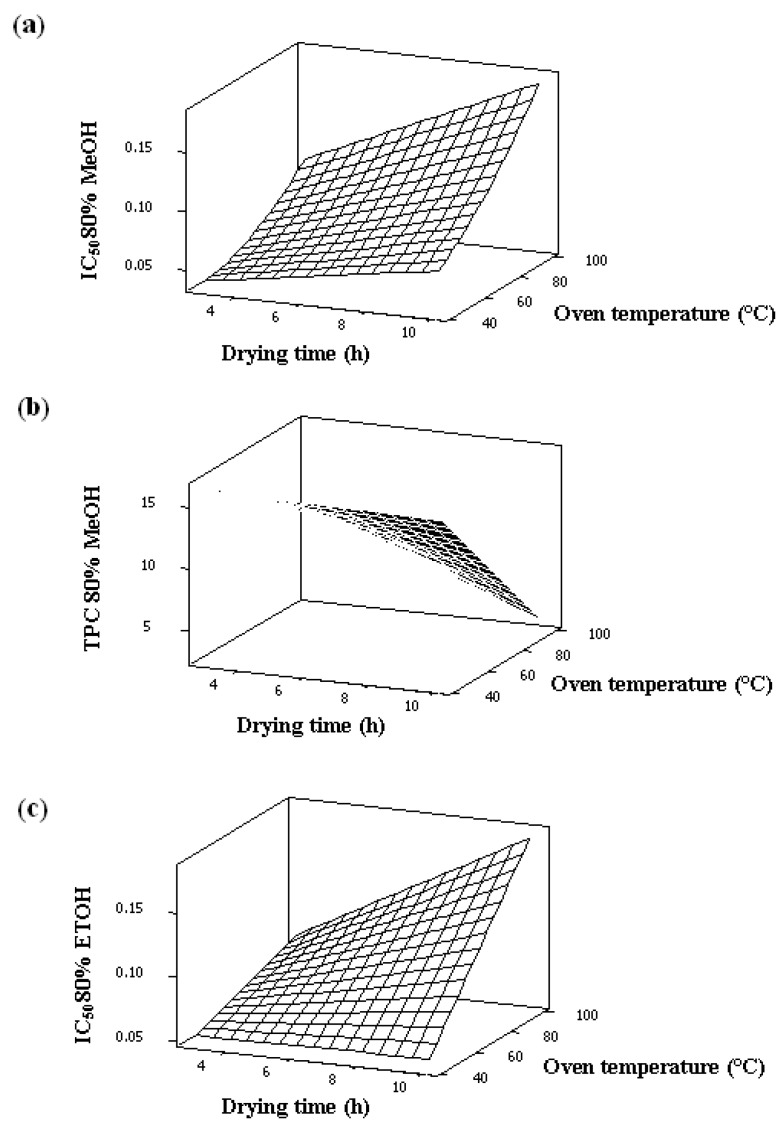
(**a**) Response surface plot of the influence of processing factors on IC_50_ 80% of methanolic extracted sample. (**b**) Response surface plot of the influence of processing factors on TPC 80% of methanolic extracted sample. (**c**) Response surface plot of the influence of processing factors on IC_50_ 80% of ethanolic extract sample.

The results indicated that a significant (*p* < 0.05) decrease in DPPH scavenging activity and TPC among the oven-dried samples extracted with the two solvents compared with the control. In Table 2 and Table 3, all responses appeared to be significantly influenced by both factors. As clearly shown in both tables, the linear effect of independent variables had a significant (*p* < 0.05) impact on all responses. The oven temperature had the greatest effect on both responses compared to drying time. Similarly, oven temperature was found to exhibit the highest significant (*p* < 0.05) influence on both DPPH and TPC for the sample extracted with the two types of solvent. The relationship between the responses and independent variables was determined by the response surface plot. From the surface plot, the increase in drying time and oven temperature caused an increase in the IC_50_ [Figure 1(a,b)] and a decrease in the TPC [Figure 1(c)] for both samples extracted with the two solvents. The shape of the surface plots in Figure 1 confirmed that independent variables exhibited an interaction effect on Y_1_, Y_2_ and Y_3_. A quadratic effect on Y_1_ and Y_4_ was observed.

### 2.5. Optimization Conditions

RSM is the most common modelling technique used to obtain desirable conditions for several variables and the impact of their interactions on responses to determine which independent variable has the maximum and minimum effects [19,20,21]. The reduction of treatment levels and experimental error are additional advantages of its use in statistics. RSM was well-utilised to optimise oven drying conditions for guava lycopene content [17]. 

The best conditions for oven temperature and drying time for DPPH radical scavenging and TPC of *C. caudatus* corresponded to the maximum value of TPC and the minimum value of IC_50_. The graphical and numerical optimisation for the determination of the most adequate operating conditions for desirable responses was performed by constructing an overlaid contour plot and response optimiser, respectively. The optimum point of sample extracted with 80% methanol was 44.5 °C for 4 h, with a predicted IC_50_ value of 0.045 mg/mL and TPC of 16.5 mg GAE/100 g DW. The oven-dried 80% ethanol extract sample exhibited the highest level of antioxidant capacity at 43.12 °C for 4.05 h with IC_50_ and TPC values of 0.055 mg/mL and 15.18 mg GAE/100 g DW, respectively. The moisture content of the optimized oven-dried *C. caudatus* was approximately 9% (w/w).

### 2.6. Validation of the Model

The adequacy of the regression equations was verified by comparing the experimental data with the predicted values obtained from the software (Table 4). The model was validated by comparing the experimental results (values) with the fitted results provided by the software as well as experimentally in the laboratory. The one sample t-test was used to compare the mean of the experimental value with the fitted values of IC_50_ and TPC. From Table 4, the results showed that the *p* value of all four t-tests were greater than 0.05, indicating a lack of significant (*p* > 0.05) differences between the experiment value and predicted value. The variance between the experimental and fitted values was negligible, and in some cases, a null value confirmed the accuracy of the study. Practical experiments at the optimum point were performed in the laboratory in triplicate, and the results were in agreement with the first values and the obtained values. This practical verification also confirmed the suitability of RSM to optimise the desirable effects of oven temperature and drying time to preserve *C. caudatus* bioactivity.

**Table 4:** Experimental and fitted value of responses and *p*-value of validation (two-sample t-test).

80% Methanol						80% Ethanol					
IC_50_ (Y_1_)			TPC (Y_2_)			IC_50_ (Y_3_)			TPC (Y_4_)		
EV^a^	FV^a^	EV-FV	EV^b^	FV^b^	EV-FV	EV^c^	FV^c^	EV-FV	EV^d^	FV^d^	EV-FV
0.074	0.072	0.002	13.82	14.191	−0.371	0.072	0.069	0.003	12.65	11.908	0.742
0.088	0.083	0.005	11.70	11.336	0.364	0.084	0.092	−0.008	10.75	10.198	0.552
0.082	0.083	−0.001	11.87	11.336	0.534	0.087	0.092	−0.005	10.10	10.198	−0.098
0.087	0.089	−0.002	10.13	10.200	−0.07	0.092	0.091	0.001	8.75	8.901	−0.151
0.142	0.144	−0.002	5.84	5.806	0.034	0.145	0.148	−0.003	5.48	6.110	−0.63
0.078	0.083	−0.005	11.32	11.336	−0.016	0.095	0.092	0.003	10.43	10.198	0.232
0.047	0.045	0.002	14.67	15.146	−0.476	0.066	0.058	0.008	14.05	14.698	−0.648
0.116	0.116	0.000	10.32	9.380	0.94	0.122	0.119	0.003	10.35	10.500	−0.15
0.088	0.087	0.001	10.82	11.271	−0.451	0.092	0.095	−0.003	9.85	10.509	−0.659
0.058	0.058	0.000	14.25	13.163	1.087	0.067	0.071	−0.004	14.94	14.446	0.494
0.054	0.057	−0.003	16.25	15.985	0.265	0.054	0.055	−0.001	12.92	13.058	−0.138
0.141	0.138	0.003	6.25	6.558	−0.308	0.142	0.135	0.007	5.34	4.859	0.481
0.091	0.087	0.004	10.43	11.271	−0.841	0.095	0.095	0.000	10.43	10.509	−0.079
0.083	0.087	−0.004	10.58	11.271	−0.691	0.093	0.095	−0.002	10.56	10.509	0.051
*p*-value = 1.000			*p*-value = 1.000			*p*-value = 0.994			*p*-value = 1.000		

Notes: EV, experimental value; FV, fitted value and EV-FV, residue; ^a,b^ Each small letters within different columns statistically not significant (*p* < 0.05; *n* = 3); Fitted values of responses were predicted by software.

### 2.7. Correlation between Antioxidant Capacity and TPC

Research concerning TPC analysis as a source of antioxidants has been demonstrated and confirmed *in vivo* and *in vitro* by many researchers. Moreover, herbal phenolic groups contribute to antioxidant power [22]. In this study, the results suggest a strong correlation between free radical scavenging activity and TPC, with *R^2^* = 83.8 and 82.5 for samples extracted with 80% methanol and 80% ethanol, respectively. This correlation might be due to the large contribution of TPC to the antioxidant capacity of *C. caudatus*. 

This finding corroborates previous research reporting a strong relationship between antioxidant capacity and TPC in an Indian medicinal plant. However, some studies have reported a weak relationship between total phenolic compounds and antioxidant capacity in plant extracts [18,23,24]. There was a lack of correlation between both variables in edible plants from Finland and wheat. The observed correlation between DPPH and TPC might be explained by the significant involvement of phenolic compounds in the antioxidant capacity of *C. caudatus*.

### 2.8. Identified Compounds in Cosmos Caudatus by HPLC

**Figure 2:**
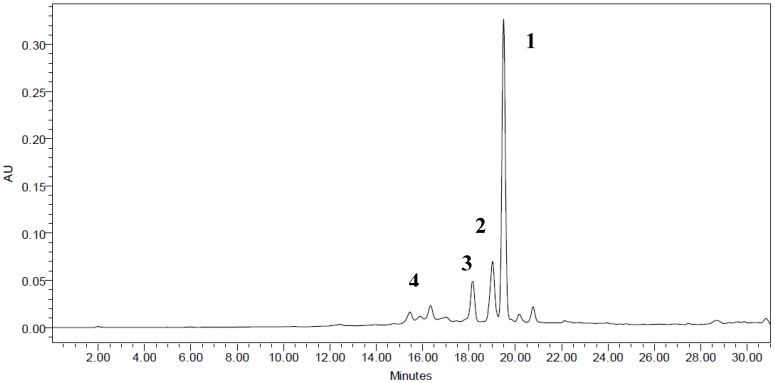
HPLC Chromatogram of oven dried *Cosmos caudatus* sample with the optimized conditions.

Flavonoids and esters are the most common secondary metabolites among phenolic compounds implicated in the antioxidant capacity of herbs. Several metabolites were identified, including quercetin rhamnoside, quercetin glucoside, rutin and chlorogenic acid (Figure 2). The major eluted peaks on the HPLC chromatograms were assigned to quercetin rhamnoside followed by quercetin glucoside, rutin and chlorogenic acid at 19.928, 19.447, 18.598 and 16.165 min, respectively (Figure 2). This finding was in agreement with previous reports on *C. caudatus* [8,9,25]. However, there were also some unidentified peaks, which might refer to other phenolic compounds.

The peak numbers as refer to the identified metabolites as follow: 1, quercetin rhamnoside; 2, quercetin glucoside; 3, rutin; 4, chlorogenic acid.

## 3. Experimental

### 3.1. Materials

The chemicals used in this study included Folin-Ciocalteau’s phenol reagent, gallic acid, sodium carbonate (Na_2_CO_3_), and 1,1-diphenyl-2-picrylhydrazyl (DPPH). In addition, 80% methanol and 80% ethanol were of analytical or HPLC grade, and all chemicals were supplied by Merck (Darmstadt, Germany). The equipment included an oven (Memmert Universal, Schwabach, Germany) and an ultrasonic bath sonicator, model 8510E-MTH (Branson, Danbury, MA, USA).

### 3.2. Plant Material

*Cosmos caudatus* samples were collected randomly from University Agriculture Park of Universiti Putra Malaysia on July 2010 directly before extraction to ensure freshness and avoid the degradation of metabolites. 

### 3.3. Sample Preparation

Samples of 200 g were used for each of the fourteen oven drying treatments, which were created using Minitab software (Version 14, Minitab Inc, State College, PA, USA) in accordance with a cubic central composite design (CCD) with the following independent parameters: drying time X_1_(4–9 h) and oven temperature X_2_ (50–90 °C) (Table 5). Drying was performed in an operating laboratory oven under maximum air velocity (forced ventilation) to avoid the effect of relative humidity on herbal compounds and drying processing and to ensure that all the treatments dried under the same humidity conditions. Dried samples from each treatment were ground into a fine powder with a laboratory blender and kept in aluminium paper in a cold room at 4 °C until further analysis. The same method was applied for the air-dried sample, which was used as a control.

**Table 5:** Coded value of independent variables.

Factor	Symbol	Coded Value				
		−1	0	1	−α	+α
Drying time (h)	X_1_	4	6.5	9	2.9645	10.0355
Oven temperature (°C)	X_2_	50	70	90	41.7157	98.2843

### 3.4. Extraction Method

The extracts used for the assays were prepared as follows: sample of each of the 14 treatments (4 g) was immersed in 80% methanol or 80% ethanol (100 mL). The mixture was shaken in a water bath sonicator at 25 °C for 1 h. This period was divided into two interval times with a break of 15 min. Next, the samples were filtered twice through analytical filter paper (Whatman number 1) in the presence of cotton tissue. The sample was rotary evaporated to remove solvent and concentrate the crude extract. Finally, the crude extract from each sample run was kept in an amber bottle to avoid exposure to light, which can cause photo-oxidation of the compounds, and stored at −4 °C in the freezer until analysis. The same extraction method was use for the air-dried sample, which served as a control. 

### 3.5. DPPH Radical Scavenging Activity

Free radical scavenging activity against 2,2-diphenyl-1-picrylhydrazyl (DPPH) radical was measured according to Chan *et al*. [11], with some modifications. Six dilutions of each treatment sample extract were prepared (6.25, 12.5, 25, 50, 100 and 200 ppm) in triplicate. Next, each extract dilution (1 mL) was placed in a test tube, followed by the addition of DPPH (2 mL, 5.9 mg/100 mL of extraction solvent). The mixture was then kept in a dark place for 30 min, and the absorbance at 517 nm was then measured in a spectrophotometer. Free radical scavenging capacity was expressed as the half maximal inhibitory concentration (IC_50_). 

### 3.6. Total Phenolic Content (TPC)

Various methods have been developed and introduced to measure TPC. In this study, TPC was measured spectrophotometrically using Folin-Ciocalteau’s reagent according to Chan *et al*. [11]. For the estimation of TPC, each treatment sample (300 µL) in triplicate was homogenised with 10-fold diluted Folin-Ciocalteau’s reagent (1.5 mL) and sodium carbonate (1.2 mL, 7.5 g/100 mL) in test tubes. The tubes were shaken and kept in the dark for 30 min before measuring the absorbance at 765 nm. TPC was expressed as mg gallic acid per 100 g of sample. The concentration of gallic acid was expressed in mg/L. 

### 3.7. Experimental Design

The effect of the two independent variables X_1_ (time of drying) and X_2_ (Oven temperature) on both IC_50_ (Y_1_, Y_3_) and TPC (Y_2_, Y_4_) was determined by applying a two- factor CCD, which was adopted to create a cubic RSM design as listed in Table 5. Furthermore, Y1 and Y3 refer to 80% methanolic extract, whereas Y2 and Y4 refer to 80% ethanolic extract. The 14 selected treatments (levels) based on CCD and the six centre points are shown in Table 1. In addition, the centre point repetition was performed six times to ensure the repeatability of the model.

### 3.8. Assortment of the Factor Ranges

In this research, a preliminary study was performed to identify the appropriate range of independent variables (drying time and oven temperature) to ensure that each treatment and their interactions are suitable. According to this preliminary study, a temperature less than 40 °C was not sufficient to dry the sample properly. However, a temperature above 100 °C could destroy and weaken the efficacy of the bioactive compounds present in the herbs. The Maillard reaction can also be caused by high temperatures [17]. Enzymatic degradation could lead to a reduction in the quality of the raw material and has some disadvantages. Therefore, ranges of oven temperature (50–90 °C) and drying time (4–9 h) were evaluated in this study.

### 3.9. Statistical Analysis

To achieve the optimum conditions that lead to the minimum loss of TPC and antioxidant capacity response surface analysis with the software Minitab 14 was used to obtain the design and analyse the data. Moreover, a suitable model was obtained by comparing the R^2^, adjusted R^2^, number of significant term, p value of regression, P value of lack of fit and error of the initial model. The TPC and IC_50_ of samples extracted with 80% methanol were analysed with full quadratic and linear interaction models. Linear interaction and linear square models were determined to be suitable for the TPC and IC_50_ of samples extracted with 80% ethanol. Furthermore, after choosing the level of variable effect on responses, non-significant coefficients (p > 0.05) were removed by performing ANOVA test. Therefore, the reduced model was applied to determine regression coefficients, the statistical meaning of model terms and regression model fitting. 

Graphical and numerical multiple optimisers were performed with the reduced model to verify the combination of factor level and optimum point and area leading to a desirable response. From the surface plot, the best visual representation of the effects of independent variables was achieved by 3D to confirm the factor interaction. The response optimiser was also adopted to provide the exact optimum points of factors. The experimental values were then compared with the fitted values to determine the accuracy of the model. All the assays were carried out in triplicate.

### 3.10. High Performance Liquid Chromatography (HPLC)

High performance liquid chromatography (HPLC) was used to identify compounds in C. caudatus samples following the procedure of Mustafa *et al*. [18] using a reverse-phase HPLC with a UV detector (Waters, Milford, MA, USA)). The Waters Xterra C18 column (4.6 mm × 150 mm, 5 µm) was coupled with the Waters C18 guard column to obtain the low dispersion required by the very fine particles. The standards used in this experiment were either previously isolated or purchased from Sigma (St. Louis, MO, USA). They included quercetin rhamnoside, quercetin glucoside, rutin and chlorogenic acid, and were selected based on a review of the previous literature. Pure methanol (solvent A) and deionized water with 0.1% trifluoroacetic acid (solvent B) were the mobile phases used. The flow rate was maintained at 0.8 mL/min. The programmed gradient was carried out using the following sequence of solvent B distributions: 80% in 0 min, 50% in 10 min, 0% in 25 min, and 80% in 30 min.

Samples and standards were prepared by dissolving the extract of the dried materials (2 mg) in 80% methanol (2 mL). The injected volumes of samples were filtered using a 0.2 µm nylon membrane into a 2-mL screw-capped sample vial. For all samples and standards, the injection was performed with a consistent volume of 20 µL by using a 1 mL injection loop. Detection was performed at wavelengths of 280 and 360 nm to monitor phenolic peaks.

## 4. Conclusions

This study was conducted to optimise oven drying method conditions (time and temperature) to obtain valuable antioxidant capacity from *C. caudatus* extracted with 80% methanol and 80% ethanol. The results indicated that 44.5 °C for 4 h and 43.12 °C for 4.05 h were the best drying time and oven temperature conditions for samples extracted with 80% methanol and 80% ethanol, respectively. Based on the optimized oven conditions, the antioxidant efficiency of *C. caudatus* extracted with 80% methanol was superior to that extracted with 80% ethanol, suggesting that the choice of solvent depends on its safety for the use of herbs as food or in research. 

The antioxidant capacity exhibited by the extract of oven-dried *C. caudatus* is high. The optimized conditions of oven-dried *C. caudatus* with maximum phytochemicals and nutritional properties could be obtained by applying RSM. This processing method might be preferable to other drying types from an economic perspective.
